# A comparative analysis of energy expenditure and substrate metabolism in male university students with overweight/obesity: Tabata vs HIIT and MICT

**DOI:** 10.3389/fendo.2024.1323093

**Published:** 2024-02-27

**Authors:** Yongbo Wang, Changming Fan, Lin Cheng, Yifei Wang, Danxing Peng, Fengcai Li, Yanbai Han, Hongli Wang

**Affiliations:** ^1^College of Physical Education and Health, Guangxi Normal University, Guilin, China; ^2^Department of Physical Education, Hebei University of Environmental Engineering, Qinhuangdao, China

**Keywords:** exercise, obesity, fat oxidation, glucose oxidation, energy expenditure

## Abstract

**Introduction:**

Exploring the energy expenditure and substrate metabolism data during exercise, 10-minute recovery, and 20-minute recovery phases in Tabata, HIIT(High-Intensity Interval Training), and MICT(Moderate-Intensity Continuous Training). This study explores the scientific aspects of weight reduction strategies, examining energy expenditure and substrate metabolism from various training perspectives. The aim is to establish a theoretical foundation for tailoring targeted exercise plans for individuals within the population with overweight/obesity.

**Methods:**

This study used an experimental design with fifteen male university students with overweight/obesity. Participants underwent random testing with Tabata, HIIT, and MICT. Tabata involved eight sets of 20 seconds exercise and 10 seconds rest, totaling 4 minutes. HIIT included four sets of power cycling: 3 minutes at 80% VO_2max_ intensity followed by 2 minutes at 20% VO_2max_. MICT comprised 30 minutes of exercise at 50% VO_2max_ intensity. Gas metabolism indices were continuously measured. Subsequently, fat and glucose oxidation rates, along with energy expenditure, were calculated for each exercise type.

**Results:**

During both the exercise and recovery phases, the Tabata group exhibited a significantly higher fat oxidation rate of (0.27 ± 0.03 g/min) compared to the HIIT group (0.20 ± 0.04 g/min, p<0.05) and the MICT group (0.20 ± 0.03g/min, p<0.001). No significant difference was observed between the HIIT and MICT groups (p=0.854). In terms of energy expenditure rate, the Tabata group maintained a substantially elevated level at 5.76 ± 0.74kcal/min compared to the HIIT group (4.81 ± 0.25kcal/min, p<0.01) and the MICT group (3.45 ± 0.25kcal/min, p<0.001). Additionally, the energy expenditure rate of the HIIT group surpassed that of the MICT group significantly (p<0.001).

**Conclusion:**

The study finds that male college students with overweight/obesity in both exercise and recovery, Tabata group has lower fat and glucose oxidation rates, and energy expenditure compared to HIIT and MICT groups. However, over the entire process, Tabata still exhibits significantly higher rates in these aspects than HIIT and MICT. Despite a shorter exercise duration, Tabata shows a noticeable “time-efficiency” advantage. Tabata can be used as an efficient short-term weight loss exercise program for male college students with overweight/obesity.

## 1 Introduction

In recent years, the improvement of living standards has brought about various health issues, with obesity being a global public health concern. The sedentary lifestyle and changes in dietary habits associated with socio-economic development have contributed to a rapid increase in global obesity rates (1). According to the World Obesity Federation’s “World Obesity Atlas 2023” report, it is projected that over the next 12 years, more than 51% of the global population—exceeding 4 billion people—will be either obese or overweight. Research indicates that obesity is a significant risk factor for chronic diseases such as cardiovascular diseases, diabetes, and musculoskeletal disorders (2). The high prevalence of obesity significantly affects the health and societal development in China, making the scientific and efficient approach to weight loss a critical issue.

Traditional weight loss exercises often involve MICT(Moderate-Intensity Continuous Training), such as walking, brisk walking, running, and cycling, which has proven to be effective (3). However, the lengthy and monotonous nature of the exercise makes it challenging for many individuals with obesity to adhere to. In recent years, HIIT(High-Intensity Interval Training) has gained attention. Initially proposed by Reindell and Roskamm (4), HIIT has become popular among fitness enthusiasts. HIIT involves repeated cycles of high-intensity exercise interspersed with low-intensity exercise or rest periods with varying recovery times (5). Compared to traditional long-duration continuous exercise, HIIT has a relatively short exercise duration but higher intensity (6, 7). HIIT is considered an exercise method that increases the metabolic rate and promotes fat consumption (8). The primary energy systems during HIIT are the phosphagen and glycolytic systems, with glucose being the main metabolic substrate. Therefore, the efficacy of HIIT in reducing fat is not solely due to the energy expenditure during exercise but also the significant aerobic demand fluctuations that enhance post-exercise metabolic rate and fat utilization (9).

However, traditional HIIT training still requires more than 20 minutes. This is where Tabata comes into play. Originating in Japan, Tabata involves 20 seconds of exercise, 10 seconds of rest, repeated for eight sets, totaling a 4-minute interval training session (10, 11). Compared to HIIT and MICT, Tabata offers advantages such as a relatively short exercise duration, high enjoyment, unrestricted space requirements, and easy implementation, making it more accessible for individuals with overweight/obesity to adhere to.

Current research primarily focuses on the effects of different exercise modalities on body composition, body components, and weight loss. Many scholars have conducted comparative studies on the fitness effects of HIIT and MICT, finding that for the same energy expenditure, HIIT yields a greater increase in VO_2max_ [23.8% vs 19.3% (12), 15.8% vs 8.0% (13), 14% vs 7% (14)]. Moreover, HIIT has been shown to reduce insulin and blood sugar levels, improve insulin sensitivity, and enhance the mechanisms of blood dynamics, metabolism, and endocrine factors involved in the pathogenesis of hypertension in healthy populations (13). The effects of HIIT are also superior to those of MICT for reducing total fat mass and abdominal fat mass, even with the same energy expenditure (15).

Physical exercise promotes browning of adipocytes, which is a transition from white adipose tissue to metabolically active brown adipose tissue. The characteristic of browning is an increase in the number of mitochondria and the dissipation of energy through heat generation, namely non shivering heat generation. Due to the large amount of energy consumed by brown adipose tissue in non shivering heat generation, this can lead to an overall increase in energy consumption. Physical exercise can promote more effective energy consumption in the body by activating these physiological processes. This is precisely why this study aims to provide new insights into the field of exercise and weight loss for obese individuals from the perspectives of energy expenditure and substrate metabolism through different exercise methods.

The debate over the superiority of weight loss effects between HIIT and MICT is a current hotspot. Although some studies suggest that HIIT is more effective for weight loss than MICT, due to differences in research methods and training programs, it is premature to conclude that HIIT is superior to MICT for weight loss (16, 17). With the emergence of Tabata, a form of exercise with shorter duration and higher intensity compared to HIIT and MICT, further research is needed to determine its potential superior effects on weight loss. Therefore, this study aims to conduct experimental research on randomly selected individuals with overweight/obesity undergoing Tabata, HIIT, and MICT. The analysis will focus on substrate metabolism and energy expenditure data during the exercise and recovery phases of 10 and 20 minutes, comparing the fat oxidation rate, glucose oxidation rate, and energy expenditure rate of the three exercise modalities. The findings aim to provide a theoretical basis for the tailored development of training plans for overweight/obese individuals.

## 2 Subjects and methods

This study employed an experimental research design involving a cohort of randomly selected fifteen male university students with overweight/obesity. Initial assessments included maximal oxygen consumption tests to determine the power corresponding to 20% VO_2max_, 50% VO_2max_, and 80% VO_2max_ intensities on a stationary bicycle. Subsequently, participants underwent random Tabata, HIIT and MICT exercise tests, each separated by a 7-day interval. Continuous measurements of gas metabolism parameters were conducted during exercise and the 10-minute and 20-minute recovery periods. This data was then utilized to calculate the fat oxidation rate, glucose oxidation rate, and energy expenditure rate for each of the three exercise modalities.

### 2.1 Participants

Fifteen male university students with overweight/obesity were randomly selected for this study (mean ± standard deviation, age: 22.85 ± 2.40 years, height: 177 ± 5.64 cm, weight: 86.40 ± 4.80 kg, Body Fat Percentage(BFP): 24.82 ± 1.43%, BMI: 27.12 ± 1.48 kg/m^2^, Fat mass:21.33 ± 2.41 kg, lean mass:64.67 ± 3.07 kg). All participants had a BMI greater than 24 kg/m^2^, according to Chinese adult BMI standards (overweight BMI > 24 kg/m^2^, obesity BMI > 28 kg/m^2^), indicating good physical condition. They reported no regular exercise habits, were non-smokers, and had no history of diabetes or metabolic disorders. Exclusion criteria included smokers, individuals with a history of diabetes or metabolic diseases. In the week preceding the experiment, participants refrained from intense physical activity, smoking, alcohol consumption, and the intake of caffeinated beverages or medications for 48 hours prior to testing. Dietary habits were recorded for the week before the experiment, focusing on normal daily meals with avoidance of high-fat and high-sugar foods. During the experiment, participants were instructed to maintain the same dietary structure and quantity as the previous week to eliminate dietary interference. Adequate sleep was encouraged, with participants advised against staying up late or having irregular sleep schedules to minimize potential impacts on the experiment. Before testing, participants were fully informed about the purpose and procedures of the study and provided informed consent.

### 2.2 Experimental control

The indoor temperature was maintained within the range of 24–26°C, with a relative humidity level between 40% and 60%. All participants underwent exercise testing precisely 2 hours postprandial, commencing at 14:30 in the afternoon. The Tabata exercise test commenced 48 hours after the conclusion of the maximal oxygen consumption test. Subsequently, the HIIT exercise test and the MICT exercise test were conducted at 7 days intervals.

### 2.3 Preliminary test

Participants in this study underwent an initial maximal oxygen consumption test. The VO_2max_ test protocol commenced with a load intensity of 20 watts, incrementing by 15 watts every two minutes. Participants were instructed to maintain a pedaling speed of 60 revolutions per minute on the stationary bicycle. The power output corresponding to 20% VO_2max_, 50% VO_2max_, and 80% VO_2max_ intensities was determined by correlating VO_2_ levels with the corresponding power output (W) achieved during the test.

### 2.4 Experimental procedures

Participants were sequentially and randomly assigned to undergo the Tabata test, HIIT test, and MICT test, with a 7-day interval between each session. On the day of testing, participants arrived at the laboratory at 14:30, where they engaged in a 20-minute seated rest period followed by a warm-up exercise. Subsequently, they commenced the exercise test after donning the cardiopulmonary testing apparatus. Post-exercise, participants continued wearing the respiratory mask, with continuous monitoring of gas metabolism parameters during the 10-minute and 20-minute recovery periods.

The Tabata test comprised four exercises (squat, jumping jacks, high knees, burpees), each lasting 20 seconds, repeated for 2 sets. After each 20-second exercise, there was a 10-second rest, resulting in a total duration of 4 minutes.

The HIIT test involved cycling at 80% VO_2max_ intensity for 3 minutes, followed by cycling at 20% VO_2max_ intensity for 2 minutes, alternating for a total of 4 sets. Participants maintained a pedal speed of 60 revolutions per minute, and the entire session lasted 20 minutes.

**Figure 1:**
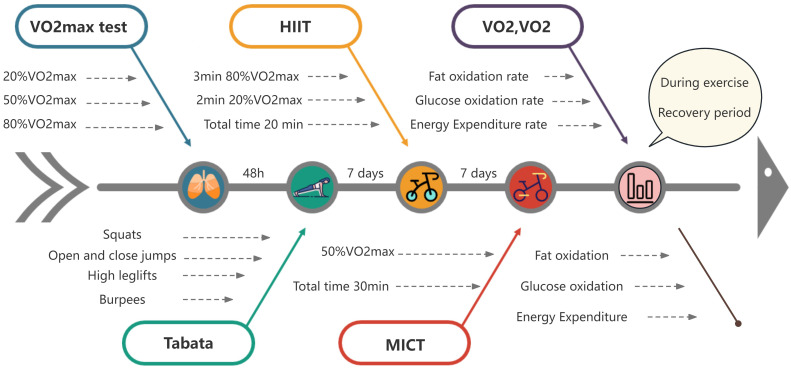
Experimental procedure chart (n=15).

For the MICT test, participants cycled at a load corresponding to 50% VO_2max_ intensity, maintaining a pedal speed of 60 revolutions per minute, for a continuous duration of 30 minutes. Refer to (**Figure 1**) for the experimental protocol.

## 3 Measurements

### 3.1 Physiological indices measurement

BMI was determined as weight/height squared (kg/m^2^). The body fat rate was measured with a body composition testerVO_2_ (L/min) and VCO_2_ (L/min) were continuously measured with the exercise cardiopulmonary tester (cosmedk5,Italy) during exercise and recovery, and VO_2max_(L/min) was completed with the instrument.It is calculated that (18, 19): ① Oxidation rate of sugar (g/min) =4.585 × VCO_2_ (L/min) -3.2256 × VO_2_ (L/min) ② Oxidation amount of sugar (g) = Oxidation rate of sugar × Time (min) ③ Fat oxidation rate (g/min) =1.695 × VO_2_ (L/min) -1.701 × VCO_2_ (L/min) ④ Oxidation amount of fat (g) = Oxidation rate of fat × Time (min) ⑤ Energy consumption rate (kcal/min) =3.716 × VO_2_ (L/min) +1.332 × VCO_2_ (L/min) ⑥ Energy consumption (kcal) =[3.716 × VO_2_ (L/min) +1.332 × VCO_2_ (L/min)] × Time (min), HR(Garmin)was recorded every 1 min throughout the test period. Rating of perceived exertion (RPE) was recorded from the beginning to the end of the exercise using a Borg scale (20).

### 3.2 Statistical analysis

G*power3.1.9.7, with an effect size of 0.25, an alpha value of 0.05, and a power of 0.80, was used to estimate the sample size.Based on the calculation, the minimum sample size necessary to satisfy the test requirements was 8. All data were statistically analyzed using SPSS 26.0 software (Chicago, IL, USA) and expressed as mean ± standard deviation. The Shapiro-Wilk test showed that the data were normally distributed. One way repeated measures ANOVA was used to analyze the fat oxidation rate, sugar consumption rate, energy consumption rate, fat oxidation amount, sugar oxidation amount, and energy consumption in Tabata, HIIT, and MICT groups during exercise, 10, and 20 minutes of recovery. Corrected by the Greenhouse–Geisser method when the sphericity test was violated. If significant differences were found, Bonferroni *post hoc* test was used to find pairwise differences. Effect sizes were reported as partial eta squared (pη^2^) for ANOVA. (pη^2^the effect of small, medium and large were 0.04, 0.25 and 0.64.) (21) Significant differences were considered at p<0.05.

## 4 Results

Energy consumption and substrate metabolism are important indicators for evaluating exercise effectiveness. Glucose and fat are important indicators of substrate metabolism, and this study mainly monitors fat oxidation rate. Glucose oxidation rate, glucose oxidation. For energy consumption, the main monitoring is the energy expenditure rate. We monitor the energy consumption and substrate metabolism analysis data of three different exercise modes at three time points: exercise period, recovery period, exercise and the entire recovery process. The purpose is to provide theoretical basis for developing targeted training plans for overweight/obese populations from the perspectives of energy consumption and substrate metabolism.

### 4.1 During exercise

**Figure 2:**
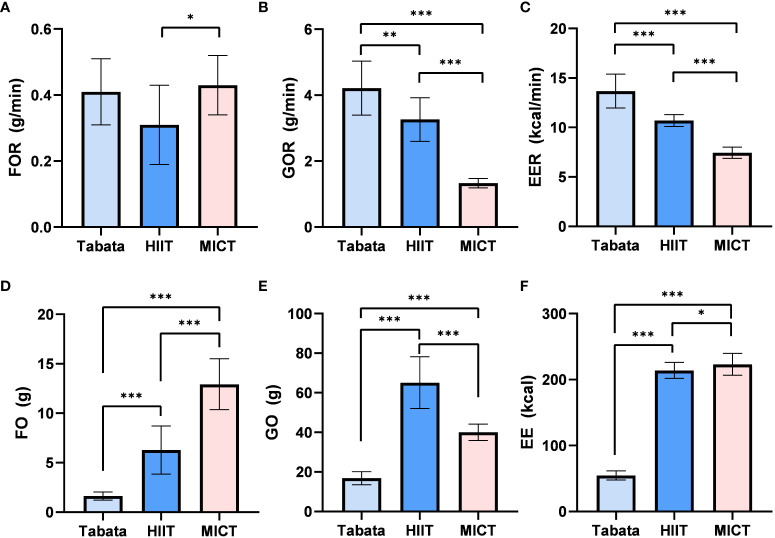
FO, Fat Oxidation; GO, Glucose Oxidation; EE, Energy Expenditure; Fat oxidation rate,Glucose Oxidation rate and Energy Expenditure rate of Tabata, HIIT and MICT during exercise [**(A–C)** n=15] Fat oxidation, Glucose Oxidation and Energy Expenditure of Tabata, HIIT and MICT during exerciese [**(D–F)** n=15] Data are expressed as mean ± SD. *p < 0.05, **p < 0.01, ***p<0.001.

During the exercise phase, there was a statistically significant difference in the fat oxidation rate among the three exercise modes (F_2,13_ = 7.215, p<0.01, pη^2^ = 0.526). The fat oxidation rate in the Tabata group (0.43 ± 0.09g/min) did not significantly differ from the HIIT group (0.34 ± 0.10g/min) or the MICT group (0.46 ± 0.08g/min) (p>0.05). However, the fat oxidation rate in the MICT group (0.46 ± 0.08g/min) was significantly higher than that in the HIIT group (0.34 ± 0.10g/min) (p<0.05) (**Figure 2A**).

There was a statistically significant difference in the glucose oxidation rate among the three exercise modes during the exercise phase (F_2,28_ = 169.501, p<0.001, pη^2^ = 0.924). The glucose oxidation rate in the Tabata group (4.31 ± 0.64g/min) was significantly higher than the HIIT group (3.28 ± 0.58g/min) (p<0.01) and the MICT group (1.30 ± 0.12g/min) (p<0.001). Additionally, the glucose oxidation rate in the HIIT group (3.28 ± 0.58g/min) was significantly higher than that in the MICT group (1.30 ± 0.12g/min) (p<0.001) (**Figure 2B**).

The energy expenditure rate during the exercise phase showed a statistically significant difference among the three exercise modes (F_2,13_ = 482.802, p<0.001, pη^2^ = 0.987). The energy expenditure rate in the Tabata group (13.67 ± 1.39kcal/min) was significantly higher than the HIIT group (10.64 ± 0.49kcal/min) and the MICT group (7.50 ± 0.46kcal/min) (p<0.001). Moreover, the energy expenditure rate in the HIIT group (10.64 ± 0.49kcal/min) was significantly higher than that in the MICT group (7.50 ± 0.46kcal/min) (p<0.001) (**Figure 2C**).

The fat oxidation amount during the exercise phase exhibited a statistically significant difference among the three exercise modes (F_2,13_ = 68.317, p<0.001, pη^2^ = 0.894). The fat oxidation amount in the Tabata group (1.65 ± 0.39g) was significantly lower than the HIIT group (6.30 ± 2.45g) (p<0.001) and the MICT group (12.87 ± 2.54g) (p<0.001). Additionally, the fat oxidation amount in the HIIT group was also significantly lower than that in the MICT group (p<0.001) (**Figure 2D**).

The glucose oxidation amount during the exercise phase demonstrated a statistically significant difference among the three exercise modes (F_2,13_ = 148.374, p<0.001, pη^2^ = 0.942). The glucose oxidation amount in the Tabata group (16.79 ± 3.31g) was significantly lower than the HIIT group (66.08 ± 13.58g) (p<0.001) and the MICT group (40.31 ± 3.79g) (p<0.001). Moreover, the glucose oxidation amount in the HIIT group was significantly higher than that in the MICT group (p<0.001) (**Figure 2E**).

The energy expenditure during the exercise phase displayed a statistically significant difference among the three exercise modes (F_2,13_ = 1572.458, p<0.001, pη^2^ = 0.989). The energy expenditure in the Tabata group (55.07 ± 6.94kcal) was significantly lower than the HIIT group (215.02 ± 11.89kcal) (p<0.001) and the MICT group (225.21 ± 17.28kcal) (p<0.001). Additionally, the energy expenditure in the HIIT group was significantly lower than that in the MICT group (p<0.05) (**Figure 2F**).

### 4.2 Recovery periods

#### 4.2.1 10-minute recovery periods

**Figure 3:**
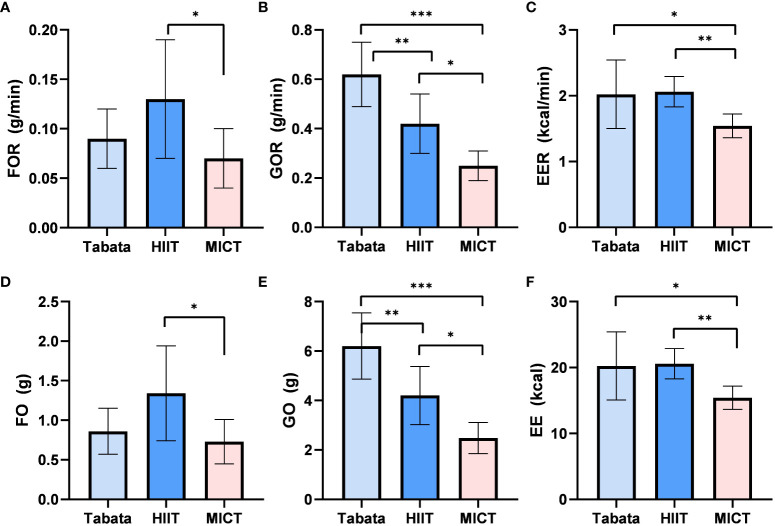
Fat oxidation rate, Glucose Oxidation rate and Energy Expenditure rate of Tabata, HIIT and MICT in 10 minutes of recovery period [**(A–C)** n=15] Fat oxidation,Glucose Oxidation and Energy Expenditure of Tabata, HIIT and MICT in 10minutes of recovery period [**(D–F)** n=15] Data are expressed as mean ± SD. *p < 0.05, **p < 0.01, ***p<0.001.

During the 10-minute recovery period, there was a statistically significant difference in fat oxidation rates among the three exercise modes (F_2,13_ = 11.210, p<0.01, pη^2^ = 0.633). The fat oxidation rate in the Tabata group (0.09 ± 0.02g/min) did not significantly differ from the HIIT group (0.13 ± 0.05g/min) or the MICT group (0.07 ± 0.02g/min) (p>0.05). However, the fat oxidation rate in the HIIT group (0.13 ± 0.05g/min) was significantly higher than that in the MICT group (0.07 ± 0.02g/min) (p<0.05) (**Figure 3A**).

There was a statistically significant difference in glucose oxidation rates among the three exercise modes during the 10-minute recovery period (F_2,28_ = 74.885, p<0.001, pη^2^ = 0.842). The glucose oxidation rate in the Tabata group (0.63 ± 0.10g/min) was significantly higher than the HIIT group (0.42 ± 0.12g/min) (p<0.01) and the MICT group (0.25 ± 0.05g/min) (p<0.001). Additionally, the glucose oxidation rate in the HIIT group (0.42 ± 0.12g/min) was significantly higher than that in the MICT group (0.25 ± 0.05g/min) (p<0.05) (**Figure 3B**).

The energy expenditure rate during the 10-minute recovery period showed a statistically significant difference among the three exercise modes (F_2,13_ = 28.436, p<0.01, pη^2^ = 0.670). The energy expenditure rate in the Tabata group (2.03 ± 0.39kcal/min) was significantly higher than the MICT group (1.53 ± 0.14kcal/min) (p<0.05) and did not significantly differ from the HIIT group (2.10 ± 0.19kcal/min) (p>0.05). Moreover, the energy expenditure rate in the HIIT group (2.10 ± 0.19kcal/min) was significantly higher than that in the MICT group (1.53 ± 0.14kcal/min) (p<0.01) (**Figure 3C**).

The fat oxidation amount during the 10-minute recovery period exhibited a statistically significant difference among the three exercise modes (F_2,13_ = 11.210, p<0.01, pη^2^ = 0.633). The fat oxidation amount in the Tabata group (0.85 ± 0.30g) did not significantly differ from the HIIT group (1.32 ± 0.50g) or the MICT group (0.72 ± 0.30g), but the HIIT group was significantly higher than the MICT group (p<0.05) (**Figure 3D**).

There was a statistically significant difference in glucose oxidation amounts among the three exercise modes during the 10-minute recovery period (F_2,13_ = 74.885, p<0.001, pη^2^ = 0.842). The glucose oxidation amount in the Tabata group (6.19 ± 1.30g) was significantly higher than the HIIT group (4.21 ± 1.21g) (p<0.01) and the MICT group (2.51 ± 0.53g) (p<0.001). Additionally, the HIIT group was significantly higher than the MICT group (p<0.05) (**Figure 3E**).

The energy expenditure during the 10-minute recovery period showed a statistically significant difference among the three exercise modes (F_2,13_ = 28.436, p<0.01, pη^2^ = 0.670). The energy expenditure in the Tabata group (20.31 ± 5.03kcal) did not significantly differ from the HIIT group (20.48 ± 2.22kcal) (p=0.83), but the Tabata group was significantly higher than the MICT group (16.21 ± 1.35kcal) (p<0.05). Moreover, the HIIT group was significantly higher than the MICT group (p<0.01) (**Figure 3F**).

#### 4.2.2 20-minute recovery periods

**Figure 4:**
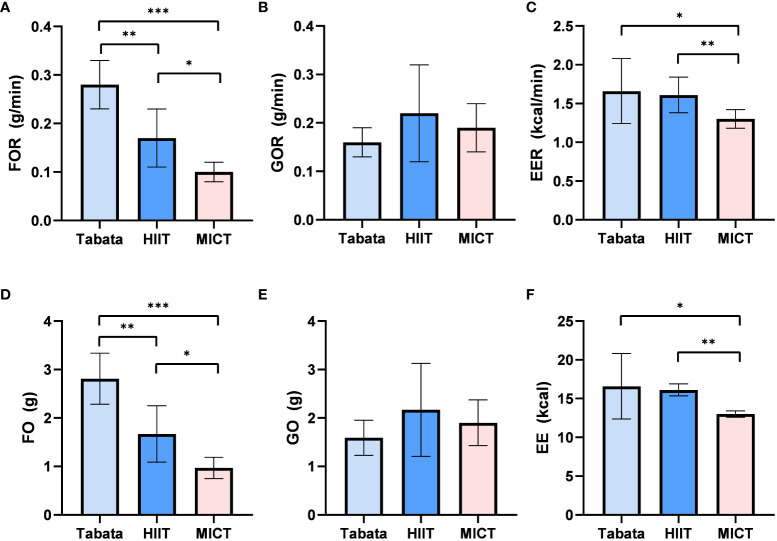
Fat oxidation rate, Glucose Oxidation rate and Energy Expenditure rate of Tabata, HIIT and MICT in 20 minutes of recovery period [**(A–C)** n=15] Fat oxidation,Glucose Oxidation and Energy Expenditure of Tabata, HIIT and MICT in 20 minutes of recovery period [**(D–F** n=15] Data are expressed as mean ± SD. *p < 0.05, **p < 0.01, ***p<0.001.

During the 20-minute recovery period, there was a statistically significant difference in fat oxidation rates among the three exercise modes (F_2,13_ = 99.948, p<0.001, pη^2^ = 0.877). The fat oxidation rate in the Tabata group (0.29 ± 0.04g/min) was significantly higher than the HIIT group (0.17 ± 0.05g/min) (p<0.01) and the MICT group (0.10 ± 0.02g/min) (p<0.001). Additionally, the fat oxidation rate in the HIIT group (0.17 ± 0.05g/min) was significantly higher than that in the MICT group (0.10 ± 0.02g/min) (p<0.05) (**Figure 4A**).

During the 20-minute recovery period, there was no significant difference in glucose oxidation rates among the three exercise modes (F_2,28_ = 3.624, p=0.083, pη^2^ = 0.652). The glucose oxidation rate in the Tabata group (0.17 ± 0.03g/min) did not significantly differ from the HIIT group (0.23 ± 0.09g/min) or the MICT group (0.19 ± 0.04g/min) (**Figure 4B**).

The energy expenditure rate during the 20-minute recovery period showed a statistically significant difference among the three exercise modes (F_2,13_ = 36.484, p<0.05, pη^2^ = 0.849). The energy expenditure rate in the Tabata group (1.67 ± 0.43kcal/min) was significantly higher than the MICT group (1.32 ± 0.10kcal/min) (p<0.05) and did not significantly differ from the HIIT group (1.60 ± 0.19kcal/min) (p>0.05). Moreover, the energy expenditure rate in the HIIT group (1.60 ± 0.19kcal/min) was significantly higher than that in the MICT group (1.32 ± 0.10kcal/min) (p<0.01) (**Figure 4C**).

The fat oxidation amount during the 20-minute recovery period exhibited a statistically significant difference among the three exercise modes (F_2,13_ = 99.948, p<0.001, pη^2^ = 0.877).The fat oxidation amount in the Tabata group (2.82 ± 0.48g) was significantly higher than the HIIT group (1.65 ± 0.54g) (p<0.01) and the MICT group (0.99 ± 0.22g) (p<0.001). Moreover, the HIIT group was significantly higher than the MICT group (p<0.05) (**Figure 4D**).

During the 20-minute recovery period, there was no significant difference in glucose oxidation amounts among the three exercise modes (F_2,13_ = 3.624, p=0.083, pη^2^ = 0.652). The glucose oxidation amount in the HIIT group (1.59 ± 0.35g) did not significantly differ from the Tabata group (2.16 ± 0.98g) or the MICT group (1.91 ± 0.47g) (**Figure 4E**).

The energy expenditure during the 20-minute recovery period showed a statistically significant difference among the three exercise modes (F_2,13_ = 36.484, p<0.05, pη^2^ = 0.849). The energy expenditure in the Tabata group (16.52 ± 4.20kcal) did not significantly differ from the HIIT group (16.09 ± 0.75kcal) (p>0.05) but was significantly higher than the MICT group (13.03 ± 0.41kcal) (p<0.05). Moreover, the HIIT group was significantly higher than the MICT group (p<0.01) (**Figure 4F**).

### 4.3 Throughout the exercise and recovery period

**Figure 5:**
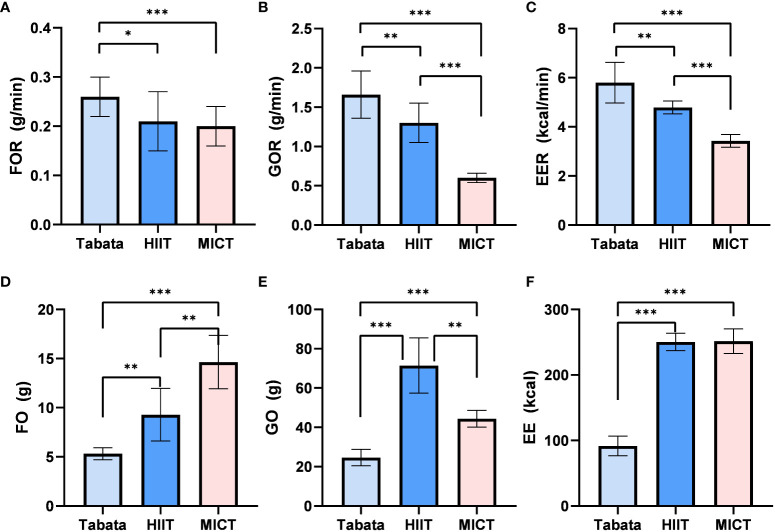
Fat oxidation rate, Glucose Oxidation rate and Energy Expenditure rate of Tabata, HIIT and MICT in the whole exercise and recovery period [**(A–C)** n=15] Fat oxidation,Glucose Oxidation and Energy Expenditure of Tabata, HIIT and MICT in the whole exercise and recovery period [**(D–F)** n=15] Data are expressed as mean ± SD. *p < 0.05, **p < 0.01, ***p<0.001.

Throughout the exercise and recovery period, there was a statistically significant difference in fat oxidation rates (F_2,28_ = 23.416, p<0.001, pη^2^ = 0.626). The overall fat oxidation rate in the Tabata group (0.27 ± 0.03g/min) was significantly higher than the HIIT group (0.20 ± 0.04g/min) (p<0.05) and the MICT group (0.20 ± 0.03g/min) (p<0.001). There was no significant difference between the HIIT group and the MICT group (p=0.854) (**Figure 5A**).

The glucose oxidation rates throughout the exercise and recovery period showed a statistically significant difference (F_2,13_ = 48.023, p<0.001, pη^2^ = 0.901). The overall glucose oxidation rate in the Tabata group (1.67 ± 0.28g/min) was significantly higher than the HIIT group (1.31 ± 0.22g/min) (p<0.01) and the MICT group (0.61 ± 0.06g/min) (p<0.001). Additionally, the HIIT group had a significantly higher glucose oxidation rate than the MICT group (p<0.001) (**Figure 5B**).

Throughout the exercise and recovery period, there was a statistically significant difference in energy expenditure rates (F_2,28_ = 89.36, p<0.001, pη^2^ = 0.962). The overall energy expenditure rate in the Tabata group (5.76 ± 0.74kcal/min) was significantly higher than the HIIT group (4.81 ± 0.25kcal/min) (p<0.01) and the MICT group (3.45 ± 0.25kcal/min) (p<0.001). Furthermore, the HIIT group had a significantly higher energy expenditure rate than the MICT group (p<0.001) (**Figure 5C**).

Throughout the exercise and recovery period, there was a statistically significant difference in fat oxidation amounts (F_2,13_ = 34.782, p<0.001, pη^2^ = 0.762). The overall fat oxidation amount in the Tabata group (5.30 ± 0.61g) was significantly lower than the HIIT group (9.34 ± 2.66g) (p<0.01) and the MICT group (14.65 ± 2.70g) (p<0.001). Additionally, the HIIT group had a significantly lower fat oxidation amount than the MICT group (p<0.01) (**Figure 5D**).

Throughout the exercise and recovery period, there was a statistically significant difference in glucose oxidation amounts (F_2,13_ = 121.423, p<0.001, pη^2^ = 0.935). The overall glucose oxidation amount in the Tabata group (24.63 ± 4.20g) was significantly lower than the HIIT group (71.50 ± 14.17g) (p<0.001) and the MICT group (45.08 ± 4.32g) (p<0.001). However, the HIIT group had a significantly higher glucose oxidation amount than the MICT group (p<0.01) (**Figure 5E**).

Throughout the exercise and recovery period, there was a statistically significant difference in overall energy expenditure (F_2,28_ = 1306.546, p<0.001, pη^2^ = 0.987). The overall energy expenditure in the Tabata group (91.48 ± 14.91kcal) was significantly lower than the HIIT group (248.32 ± 13.32kcal) (p<0.001) and the MICT group (252.07 ± 19.02kcal) (p<0.001). However, there was no significant difference in energy expenditure between the HIIT group and the MICT group (**Figure 5F**).

### 4.4 Heart rate and rating of perceived exertion

During the exercise period, there was a statistically significant difference in heart rate among the three exercise modes (F_2,28_ = 322.533, p<0.001). In the 10-minute recovery period, there was a statistically significant difference in heart rate among the three exercise modes (F_2,28_ = 102.743, p<0.001). Similarly, during the 20-minute recovery period, there was a statistically significant difference in heart rate among the three exercise modes (F_2,28_ = 86.672, p<0.001) (**Table 1**).

**Table 1:** Heart rate responses over the entire trial (beats/min).

	During exercise	T_10_	T_20_
Tabata	173.80 ± 4.31	117.12 ± 5.20	98.73 ± 4.03
HIIT	158.53 ± 6.62	103.27 ± 7.92	91.31 ± 5.47
MICT	121.33 ± 6.01	93.42 ± 6.02	80.72 ± 3.65
p value	p<0.001	p<0.001	p<0.001

Data are mean ± standard deviation (n =15). T_10_, 10 minutes of recovery period; T_20_, 20 minutes of recovery period.

Throughout the exercise period, there was a statistically significant difference RPE among the three exercise modes (F_2,28_ = 147.83, p<0.001). In the 10-minute recovery period, there was a statistically significant difference in RPE among the three exercise modes (F_2,28_ = 84.721, p<0.001). Additionally, during the 20-minute recovery period, there was a statistically significant difference in RPE among the three exercise modes (F_2,28_ = 93.472, p<0.001) (**Table 2**).

**Table 2:** Changes in RPE during exercise in three groups.

	During exercise	T_10_	T_20_
Tabata	17.38 ± 0.56	11.82 ± 0.53	10.04 ± 0.68
HIIT	15.01 ± 0.62	10.21 ± 1.01	9.41 ± 0.38
MICT	12.30 ± 1.42	9.45 ± 0.27	8.34 ± 0.40
p value	p<0.001	p<0.001	p<0.001

Data are mean ± standard deviation (n = 15). T_10_, 10 minutes of recovery period; T_20_, 20 minutes of recovery period.

## 5 Discussion

### 5.1 Analysis of substrate metabolism and energy expenditure characteristics during Tabata, HIIT, and MICT

The findings of this study reveal that, during exercise, the MICT group exhibits a significantly higher fat oxidation quantity compared to the Tabata and HIIT groups. However, the fat oxidation rate of the MICT group only surpasses that of the HIIT group, and there is no significant difference in fat oxidation rates between the Tabata and MICT groups. The study suggests (22) that the duration of exercise significantly influences the body’s ratio of glucose to lipid energy provisioning. The possible explanation lies in the relatively stable state of the body during MICT exercise at 50% VO_2max_ intensity. This steadiness allows the body’s oxygen intake to precisely match the required oxygen quantity, gradually promoting energy conservation primarily through the utilization of fat. It is speculated that this phenomenon signifies the initiation of the body’s energy-saving function. The reasons behind this may be linked to the gradual depletion of muscle glycogen, reduced glucose stores, leading to decreased levels of adrenaline and insulin, consequently enhancing the rate of fat consumption.

In terms of sugar metabolism, the HIIT group demonstrates a significantly higher glucose oxidation quantity than the Tabata and MICT groups. However, the sugar consumption rate in Tabata is notably higher than in the other two groups. Exercise intensity influences the efficiency of sugar oxidation during exercise, and as the intensity increases, so does the efficiency of sugar oxidation metabolism (23). High-intensity exercise results in rapid glycogen consumption. For instance, in 1 minute of exercise at 150% VO_2max_ intensity, muscle glycogen levels can decrease by 20% (24). Conversely, a set of high-intensity intermittent exercises can reduce glycogen levels to 28%-37% of pre-exercise levels (24, 25). The energy consumption during exercise is significantly higher in the MICT group compared to the Tabata and HIIT groups. However, Tabata’s energy consumption rate is also significantly higher than the other two groups. Moderate-intensity continuous exercise, due to its lower intensity, is easier for subjects to sustain, resulting in higher energy consumption and fat oxidation rates. This proves beneficial in preventing cardiovascular diseases, obesity, diabetes, and reducing overall mortality rates (26–29).

At the same time, body composition has a significant impact on energy expenditure and substrate metabolism. Research has found that body weight has a significant impact on sugar and total energy expenditure, but has no significant effect on the proportion of fat and substrate metabolism energy supply. The reason may be that different body fat levels and body weight have a certain impact on exercise ability, with the greatest impact on defatted weight. Defatted weight is highly correlated with the aerobic and anaerobic capacity of the human body, and there is a significant difference in defatted weight between weights. Therefore, when sugar is needed for rapid oxidation energy supply, there is a significant difference in weight between weights. Gao Binghong’s research found that there is a high correlation between the defatted weight, muscle weight, and anaerobic capacity of judo athletes, with a correlation coefficient between 0.66 and 0.9. It can be considered that differences in body fat levels seriously affect substrate metabolism and energy consumption.

### 5.2 Substrate metabolism and energy expenditure analysis during the recovery period of Tabata, HIIT, and MICT

At the 10-minute recovery mark, the HIIT group exhibits a significantly higher fat oxidation rate and quantity than the MICT group, with no significant difference compared to the Tabata group. Regarding sugar metabolism, the Tabata group shows a significantly higher sugar oxidation rate and quantity than both the HIIT and MICT groups. The MICT group demonstrates significantly lower energy consumption and energy consumption rates compared to the other two groups. At the 20-minute recovery stage, the Tabata group’s fat oxidation rate and quantity are significantly higher than the other two groups. In terms of sugar metabolism, there are no significant differences among the three groups. Energy consumption induced by exercise includes not only during but also post-exercise excess post-exercise energy expenditure (EPEE). This study’s results indicate that while energy consumption during Tabata and HIIT exercises is significantly lower than MICT, the energy consumption during the recovery period is significantly higher than MICT. This aligns with previous research findings (30–32). With increasing exercise intensity, post-exercise sugar oxidation rates and quantities gradually decrease, while fat oxidation rates, quantities, and overall energy consumption increase. This is primarily due to the maintenance of high levels of endocrine hormones, body temperature, and pulmonary ventilation after high-intensity exercise. Oxygen reserves need rapid replenishment, the ATP-CP system requires quick supplementation, and lactate needs swift clearance. At this stage, the primary energy supply shifts from anaerobic to aerobic, with the main substance for aerobic energy supply transitioning from sugar to fat. post-exercise blood levels of free fatty acids significantly increase. Research demonstrates a correlation between the percentage of fat energy supply after exercise and exercise intensity and duration. Exercise intensity affects the amount of excess post-exercise oxygen consumption, while exercise duration extends the time of excess post-exercise oxygen consumption (33, 34). There is currently limited research on the recovery to resting state after high-intensity intermittent exercise, and changes in substrate levels can reflect changes in fat metabolism. Studies show that completing high-intensity intermittent exercise before eating results in a significantly greater decrease in total cholesterol and very low-density lipoprotein levels compared to the MICT group (35–37), demonstrating the superior effect of high-intensity intermittent exercise in enhancing post-exercise fat metabolism. This study confirms that Tabata significantly enhances post-exercise fat metabolism during the recovery period, surpassing both HIIT and MICT.

The fat mass of male college students with overweight/obesity is relatively higher than that of ordinary college students. Due to the higher oxidation and energy supply ratio of fat after high-intensity interval exercise, and the increase in exercise intensity, male college students with overweight/obesity can use high-intensity interval exercise as a supplement to play the role of excessive oxygen consumption after exercise.

### 5.3 Substrate metabolism and energy expenditure characteristics of Tabata, HIIT, and MICT during the entire exercise and recovery process

The results of this study indicate that throughout the exercise and recovery period, the fat oxidation rate of the Tabata group is significantly higher than that of the HIIT and MICT groups, with no significant difference between the HIIT and MICT groups. However, the fat oxidation quantity of the MICT group is significantly higher than the other two groups during the entire process. This is attributed to the short duration of Tabata exercises, lasting only 4 minutes. Considering that Tabata is 16 minutes shorter than HIIT and 26 minutes shorter than MICT, there is also a corresponding resting fat oxidation during this period, and the fat oxidation rate of Tabata is higher than the other two groups. Therefore, in reality, the difference in fat oxidation quantity of the Tabata group should be less. Combining the entire exercise and recovery period, it is found that, in terms of fat burning effectiveness, Tabata outperforms the MICT group, and the MICT group surpasses the HIIT group. In terms of overall sugar metabolism during the exercise and recovery period, both Tabata and HIIT show significantly higher sugar oxidation rates than MICT. Although the MICT exercise duration is 10 minutes longer than HIIT, the sugar oxidation quantity of HIIT is still significantly higher than MICT. The conversion of energy supply from anaerobic to aerobic in HIIT, characterized by the continuous repetition of cycles, may lead to a high dependence on sugar oxidation energy supply and a high proportion of aerobic oxidation energy of glycogen in HIIT. The changes in substrate metabolism during Tabata exercises and the recovery period may be related to changes in the quantity and activity of skeletal muscle fat metabolism-related enzymes induced by HIIT (38, 39), enhancing fat metabolism efficiency. Additionally, HIIT can rapidly deplete glycogen in a short period (40). The post-exercise glycogen compensation mechanism may induce a tendency for glycogen to be resynthesized after exercise and fat metabolism to be characterized by decomposition, oxidation, and energy supply. This study also found that, although the exercise duration of HIIT is shorter than MICT, the total energy consumption during the “exercise + recovery period” induced by exercise is not significantly different between HIIT and MICT. Although the energy consumption of the Tabata group is significantly lower than the other two groups, this is because of the shorter exercise duration compared to the other two groups. However, the energy consumption rate is still significantly higher than the HIIT and MICT groups. The direct reason for this phenomenon may be that Tabata and HIIT have a deeper impact on EPEE (energy consumption rates at 10 and 20 minutes during recovery are significantly higher than MICT). HIIT achieves the total energy consumption of a longer MICT exercise duration with less exercise time (41, 42), and Tabata has a shorter time compared to HIIT and MICT, with higher fat oxidation rates and energy consumption rates. It may be more suitable for individuals who don’t have time for long-duration low-intensity aerobic exercise.

## 6 Conclusion

This study found that male college students with overweight/obesity in the Tabata group had lower levels of fat oxidation, sugar oxidation, and energy expenditure throughout the exercise and recovery periods compared to the HIIT and MICT groups. However, during the entire process, the rates of fat oxidation, sugar oxidation, and energy consumption were significantly higher in the HIIT and MICT groups, and Tabata exercise time was significantly shorter than the other two groups, demonstrating a certain “time efficiency” advantage. Tabata can be used as an efficient short-term weight loss exercise program for male college students with overweight/obesity.

## Data availability statement

The raw data supporting the conclusions of this article will be made available by the authors, without undue reservation.

## Ethics statement

The studies involving humans were approved by ethical review of the Human Ethics Committee of Guangxi Normal University. Ethical review number: 20231129001. The studies were conducted in accordance with the local legislation and institutional requirements. The participants provided their written informed consent to participate in this study.

## Author contributions

YBW: Writing – original draft. MCF: Writing – review & editing. LC: Writing – review & editing. YFW: Writing – review & editing. XDP: Writing – review & editing. CFL: Writing – review & editing. BYH: Writing – review & editing. LHW: Writing – review & editing.

## References

[1] Hassan B Farooqui SI Khan MU Farhad A Adnan QUA. Effects of mode and duration of exercises on fat mass of obese population. J Coll Physicians Surg Pak. (2020) 30(4):412–6. doi: 10.29271/jcpsp.2020.04.412 32513363

[2] Elagizi A Kachur S Carbone S Lavie CJ Blair SN. A review of obesity, physical activity, and cardiovascular disease. Curr Obes Rep. (2020) 9(4):571–81. doi: 10.1007/s13679-020-00403-z 32870465

[3] Valkenborghs SR van Vliet P Nilsson M Zalewska K Visser MM Erickson KI. Aerobic exercise and consecutive task-specific training (AExaCTT) for upper limb recovery after stroke: A randomized controlled pilot study. Physiother Res Int. (2019) 24(3):1775. doi: 10.1002/pri.1775 30942552

[4] Billat LV. Interval training for performance: A scientific and empirical practice. special recommendations for middle- and long-distance running. Part I: Aerobic Interval Training Sports Med. (2001) 31:13–31. doi: 10.2165/00007256-200131010-00002 11219499

[5] Cadenas-Sanchez C Fernández-Rodríguez R Martínez-Vizcaíno V de Los Reyes González N Lavie CJ Galán-Mercant A. A systematic review and cluster analysis approach of 103 studies of high-intensity interval training on cardiorespiratory fitness. Eur J Prev Cardiol. (2023) 21:309. doi: 10.1093/eurjpc/zwad309 37738464

[6] Yin M Chen Z Nassis GP Liu H Li H Deng J. Chronic high-intensity interval training and moderate-intensity continuous training are both effective in increasing maximum fat oxidation during exercise in overweight and obese adults: A meta-analysis. J Exerc Sci Fit. (2023) 21(4):354–65. doi: 10.1016/j.jesf.2023.08.001 PMC10494468 37701124

[7] Gilson ND Andersson D Papinczak ZE Rutherford Z John J Coombes JS. High intensity and sprint interval training, and work-related cognitive function in adults: A systematic review. Scand J Med Sci Sports. (2023) 33(6):814–33. doi: 10.1111/sms.14349 36916717

[8] Martins C Kazakova I Ludviksen M Mehus I Wisloff U Kulseng B. High-intensity interval training and isocaloric moderate-intensity continuous training result in similar improvements in body domposition and fitness in obese individuals. Int J Sport Nutr Exerc Metab. (2016) 26(3):197–204. doi: 10.1123/ijsnem.2015-0078 26479856

[9] Keating SE Johnson NA Mielke GI Coombes JS. A systematic review and meta-analysis of interval training versus moderate-intensity continuous training on body adiposity. Obes Rev. (2017) 18(8):943–64. doi: 10.1111/obr.12536 28513103

[10] Lee KJ Noh B An KO. Impact of synchronous online physical education classes using tabata training on adolescents during COVID-19: a randomized controlled study. Int J Environ Res Public Health. (2021) 18(19):10305. doi: 10.3390/ijerph181910305 34639604 PMC8507984

[11] Tabata I. Tabata training: one of the most energetically effective high-intensity intermittent training methods. J Physiol Sci. (2019) 69(4):559–72. doi: 10.1007/s12576-019-00676-7 PMC10717222 31004287

[12] Trapp EG Chisholm DJ Freund J Boutcher SH. The effects of high-intensity intermittent exercise training on fat loss and fasting insulin levels of young women. Int J Obes (Lond). (2008) 32(4):684–91. doi: 10.1038/sj.ijo.0803781 18197184

[13] Ciolac EG Bocchi EA Bortolotto LA Carvalho VO Greve JM Guimarães GV. Effects of high-intensity aerobic interval training vs. moderate exercise on hemodynamic, metabolic and neuro-humoral abnormalities of young normotensive women at high familial risk for hypertension. Hypertens Res. (2010) 33(8):836–43. doi: 10.1038/hr.2010.72 20448634

[14] Nybo L Sundstrup E Jakobsen MD Mohr M Hornstrup T Simonsen L. High-intensity training versus traditional exercise interventions for promoting health. Med Sci Sports Exerc. (2010) 42(10):1951–8. doi: 10.1249/MSS.0b013e3181d99203 20195181

[15] Kolnes KJ Petersen MH Lien-Iversen T Højlund K Jensen J. Effect of exercise training on fat loss-energetic perspectives and the role of improved adipose tissue function and body fat distribution. Front Physiol. (2021) 24:737709(12). doi: 10.3389/fphys.2021.737709 PMC8497689 34630157

[16] Keating SE Johnson NA Mielke GI Coombes JS. A systematic review and meta-analysis of interval training versus moderate-intensity continuous training on body adiposity. Obes Rev. (2017) 18(8):943–64. doi: 10.1111/obr.12536 28513103

[17] Guo Z Li M Cai J Gong W Liu Y Liu Z. Effect of high-intensity interval training vs. moderate-intensity continuous training on fat loss and cardiorespiratory fitness in the young and middle-aged a systematic review and meta-analysis. Int J Environ Res Public Health. (2023) 20(6):4741. doi: 10.3390/ijerph20064741 36981649 PMC10048683

[18] Trombold JR Christmas KM Machin DR Kim IY Coyle EF. Acute high-intensity endurance exercise is more effective than moderate-intensity exercise for attenuation of postprandial triglyceride elevation. J Appl Physiol. (1985) 114(6):792–800. doi: 10.1152/japplphysiol.01028.2012 23372145

[19] Frayn KN. Calculation of substrate oxidation rates in vivo from gaseous exchange. J Appl Physiol. (1983) 55:628–34. doi: 10.1152/jappl.1983.55.2.628 6618956

[20] Borg GA. Psychophysical bases of perceived exertion. Med Sci Sports Exerc. (1982) 14:377–81. doi: 10.1249/00005768-198205000-00012 7154893

[21] Ferguson CJ. An effect size primer: A guide for clinicians and researchers. Prof Psychology: Res Pract. (2009) 40:532–8. doi: 10.1037/a0015808

[22] Ronsen O Haug E Pedersen BK Bahr R. Increased neuroendocrine response to a repeated bout of endurance exercise. Med Sci Sports Exerc. (2001) 33(4):568–75. doi: 10.1097/00005768-200104000-00010 11283432

[23] Brun JF Myzia J Varlet-Marie E Raynaud de Mauverger E Mercier J. Beyond the calorie paradigm: taking into account in practice the balance of fat and carbohydrate oxidation during exercise? Nutrients. (2022) 14(8):1605. doi: 10.3390/nu14081605 35458167 PMC9027421

[24] Noakes TD Prins PJ Volek JS D'Agostino DP Koutnik AP. Low carbohydrate high fat ketogenic diets on the exercise crossover point and glucose homeostasis. Front Physiol. (2023) 14:1150265. doi: 10.3389/fphys.2023.1150265 37057184 PMC10086139

[25] Prins PJ Noakes TD Buga A D'Agostino DP Volek JS Buxton JD. Low and high carbohydrate isocaloric diets on performance, fat oxidation, glucose and cardiometabolic health in middle age males. Front Nutr. (2023) 10:1084021. doi: 10.3389/fnut.2023.1084021 36845048 PMC9946985

[26] Wewege MA Thom JM Rye KA Parmenter BJ. Aerobic, resistance or combined training: a systematic review and meta-analysis of exercise to reduce cardiovascular risk in adults with metabolic syndrome. Atherosclerosis. (2018) 274:162–71. doi: 10.1016/j.atherosclerosis.2018.05.002 29783064

[27] Chipkin SR Klugh SA Chasan-Taber L. Exercise and diabetes. Cardiol Clin. (2001) 19(3):489–505. doi: 10.1016/s0733-8651(05)70231-9 11570119

[28] Zanuso S Jimenez A Pugliese G Corigliano G Balducci S. Exercise for the management of type 2 diabetes: a review of the evidence. Acta Diabetol. (2010) 47(1):15–22. doi: 10.1007/s00592-009-0126-3 19495557

[29] Yang Z CA S Mao C Tang J Farmer AJ. Resistance exercise versus aerobic exercise for type 2 diabetes: a systematic review and meta-analysis. Sports Med. (2014) 44(4):487–99. doi: 10.1007/s40279-013-0128-8 24297743

[30] Lu Y Wiltshire HD Baker JS Wang Q Ying S. The effect of tabata-style functional high-intensity interval training on cardiometabolic health and physical activity in female university students. Front Physiol. (2023) 14:1095315. doi: 10.3389/fphys.2023.1095315 36923290 PMC10008870

[31] Schaun GZ Alberton CL Ribeiro DO Pinto SS. Acute effects of high-intensity interval training and moderate-intensity continuous training sessions on cardiorespiratory parameters in healthy young men. Eur J Appl Physiol. (2017) 117(7):1437–44. doi: 10.1007/s00421-017-3636-7 28488137

[32] Matsuo T Ohkawara K Seino S Shimojo N Yamada S Ohshima H. An exercise protocol designed to control energy expenditure for long-term space missions. Aviat Space Environ Med. (2012) 83(8):783–9. doi: 10.3357/asem.3298.2012 22872993

[33] Moon J Oh M Kim S Lee K Lee J Song Y. Intelligent estimation of exercise induced energy expenditure including excess post-exercise oxygen consumption (EPOC) with different exercise intensity. Sensors (Basel). (2023) 23(22):9235. doi: 10.3390/s23229235 38005621 PMC10675648

[34] McCarthy SF Jarosz C Ferguson EJ Kenno KA Hazell TJ. Intense interval exercise induces greater changes in post-exercise metabolism compared to submaximal exercise in middle-aged adults. Eur J Appl Physiol. (2023) 11:1–6. doi: 10.1007/s00421-023-05334-w 37819613

[35] Maylor BD Zakrzewski-Fruer JK Orton CJ Bailey DP. Beneficial postprandial lipaemic effects of interrupting sedentary time with high-intensity physical activity versus a continuous moderate-intensity physical activity bout: a randomised crossover trial. J Sci Med Sport. (2018) 21(12):1250–5. doi: 10.1016/j.jsams.2018.05.022 29895406

[36] Gabriel B Ratkevicius A Gray P Frenneaux MP Gray SR. High-intensity exercise attenuates postprandial lipaemia and markers of oxidative stress. Clin Sci (Lond). (2012) 123(5):313–21. doi: 10.1042/CS20110600 22435779

[37] Vitale K Getzin A. Nutrition and supplement update for the endurance athlete: review and recommendations. Nutrients. (2019) 11(6):1289. doi: 10.3390/nu11061289 31181616 PMC6628334

[38] Bellou E Magkos F Kouka T Bouchalaki E Sklaveniti D Maraki M. Effect of high-intensity interval exercise on basal triglyceride metabolism in non-obese men. Appl Physiol Nutr Metab. (2013) 38(8):823–9. doi: 10.1139/apnm-2012-0468 23855269

[39] Mengeste AM Rustan AC Lund J. Skeletal muscle energy metabolism in obesity. Obes (Silver Spring). (2021) 29(10):1582–95. doi: 10.1002/oby.23227 34464025

[40] Frampton J Murphy KG Frost G Chambers ES. Short-chain fatty acids as potential regulators of skeletal muscle metabolism and function. Nat Metab. (2020) 2(9):840–8. doi: 10.1038/s42255-020-0188-7 32694821

[41] Panissa VLG Fukuda DH Staibano V Marques M Franchini E. Magnitude and duration of excess of post-exercise oxygen consumption between high-intensity interval and moderate-intensity continuous exercise: A systematic review. Obes Rev. (2021) 22(1):13099. doi: 10.1111/obr.13099 32656951

[42] Moniz SC Islam H Hazell TJ. Mechanistic and methodological perspectives on the impact of intense interval training on post-exercise metabolism. Scand J Med Sci Sports. (2020) 30(4):638–51. doi: 10.1111/sms.13610 31830334

